# Prevalence of vitamin D deficiency in pregnant women and their babies in Bhaktapur, Nepal

**DOI:** 10.1186/s40795-019-0294-7

**Published:** 2019-05-29

**Authors:** Dhruba Shrestha, Saraswati Budhathoki, Sabi Pokhrel, Ashok Kumar Sah, Raj Kumar Shrestha, Ganendra Bhakta Raya, Reena Shrestha, Rasila Pasakhala, Christopher Smith, Bhim Gopal Dhoubhadel

**Affiliations:** 1 Department of Paediatrics, Siddhi Memorial Hospital, Bhaktapur, Nepal; 2 School of Tropical Medicine and Global Health (TMGH), Nagasaki University, Nagasaki, Japan

**Keywords:** Vitamin D, Calcium, Pregnancy, Newborn, Nutrition, Deficiency, Supplementation, Nepal, Asia

## Abstract

**Background:**

Vitamin D deficiency has been observed worldwide in pregnant women and their newborns. Maternal vitamin D deficiency can lead to deficiency in their newborn baby and has been linked with various complications during pregnancy and delivery. There is risk of premature delivery and it is associated with high neonatal mortality.

**Methods:**

Seventy-nine pregnant women who were admitted to the Siddhi Memorial Hospital for delivery and their newborn babies were enrolled in the study. Maternal blood samples were taken before delivery while umbilical cord blood samples of their babies were taken after delivery. Serum vitamin D level and calcium level were assessed by fluorescence immunoassay using Ichromax vitamin D kit and endpoint method, respectively in the Siddhi Memorial Hospital laboratory.

**Results:**

Mean +/− SD serum vitamin D and calcium levels in pregnant mother before delivery were 14.6 +/− 8.5 ng/ml and 8.0 +/− 0.5 mg/dl, respectively, and in the cord blood were 25.7 +/− 11.2 ng/ml and 8.6 +/− 0.9 mg/dl, respectively. Eighty-one percent of the mothers and 35.8% of their babies were found to have vitamin D deficiency. Although 97.5% of the pregnant women were taking calcium supplementation, serum calcium was found lower than the normal reference value in 67% of the pregnant women and 64.2% of their babies. There were a linear relationship between the maternal and baby’s serum vitamin D (*P* < 0.001) and calcium (*P* < 0.001) levels.

**Conclusion:**

There is high prevalence of vitamin D and calcium deficiency in pregnant mothers and newborn babies in Bhaktapur, Nepal. Pregnant women need to be supplemented with adequate amounts of these nutrients.

## Background

Vitamin D deficiency is observed worldwide in pregnant women and their neonates [1–5]. Maternal vitamin D deficiency is associated with various problems in their babies such as preterm delivery, low birth weight, neonatal hypocalcemia etc. that can be associated with neonatal deaths [6].

Vitamin D deficiency can be present in people who have a vegetarian diet, who are not adequately exposed to sunlight, or consume a low amount of dairy products. Vitamin D is transferred to the fetus from mother. Vitamin D deficiency in mother can lead to deficiency in the newborn baby. Vitamin D and calcium requirements during pregnancy are higher than the normal recommended dose. But the exact dose of calcium and vitamin D supplementation is still debatable [7]. Human milk contains 15–50 IU/L of vitamin D which is inadequate to fulfill the daily requirement of the growing baby [8]. Hence exclusively breastfed babies might be at risk of vitamin D deficiency. The American Academy of Pediatrics (AAP) has recommended to supplement all exclusively breast fed neonates with 200 IU/day of vitamin D3 to prevent adverse events due to vitamin D deficiency [9].

Studies conducted in south-east Asian region have shown people living in this area are prone to vitamin D deficiency [10, 11], and its prevalence in some areas is more than 80% [12–14]. To our knowledge, vitamin D status in pregnant women and their newborn babies in Nepal has not been studied and the country is in high prone area. In this study we examine the prevalence of vitamin D and calcium deficiency among pregnant women and their babies in Bhaktapur, Nepal.

## Methods

### Study design and setting

This is a cross-sectional study conducted from November 2017 to April 2018 in Siddhi Memorial Hospital, located in Bhaktapur, Nepal; a local non-governmental maternal and children hospital that provides specialized services to pregnant mothers and children in the district.

### Enrollment, sample collection and testing for vitamin D and calcium

All pregnant women who were admitted to the hospital during the study period were approached for the study. Women who denied to participate in the study or had some medical complications such as pregnancy induced hypertension; gestational diabetes mellitus and hypothyroidism were excluded from the enrollment in the study. After counseling and informed consent the eligible women were enrolled in the study. Five ml of venous blood sample was taken for the laboratory test of vitamin D and calcium.

After delivery of a baby, cord was clamped and cut. Five ml of cord blood was obtained from the segment attached to the placenta before the placenta was delivered. Both the blood samples of mother and baby were processed and serum was collected in Siddhi Memorial Hospital laboratory. The serum was then tested for serum vitamin D level and calcium level. 25-Hydroxyvitamin D (25(OH)D2/D3) was analyzed by fluorescence immunoassay using Ichromax Vitamin D kit (Boditech Med Inc., Korea). The assay had the coefficient of variation (CV) < 10% and comparability 0.954 for the detection of vitamin D in serum. Total serum calcium level was analyzed by using o-Cresolphthalein Complexone (o-CPC) method (Accurex Biomedical Pvt. Ltd., India).

### Data collection and statistical analysis

Demographic and clinical data were first collected in a standardized questionnaire in a paper form which were later transferred into Epi Info 7 software (Version no. 7.2.1, CDC, Atlanta, USA). Statistical analyses were performed using STATA 14 (StataCorp, Texas, USA). Continuous variables were presented as means and the standard deviation (SD), and categorical variables as proportions (%). The serum level of vitamin D and calcium were categorized as shown in Table 1. Linear regression was performed in order to look for the association between maternal and cord blood vitamin D and calcium level. Multivariate linear regression was performed to adjust possible confounding factors: sex, gestational age, and birth weight. *P* value of less than 0.05 was taken as significant.

**Table 1:** Reference range of vitamin D and calcium

Status	ng/ml (nmol/L)
Vitamin D [29]	
Deficiency	< 20 (< 50)
Insufficiency	20–29 (50–75)
Sufficiency	30–100 (75–250)
Potential toxicity	> 100 (> 250)
	mg/dl (mmol/L)
Total serum calcium	
Cord blood	9.0–11.5 (2.2–2.8)
Adult	8.4–10.2 (2.1–2.5)

### Ethical consideration

Informed written consent was taken from the admitted mother before enrolling in the study and testing for blood samples for vitamin D and calcium. The research was approved by Nepal Health Research Council (NHRC), Kathmandu (Registration number: 329/2017).

## Results

**Fig. 1:**
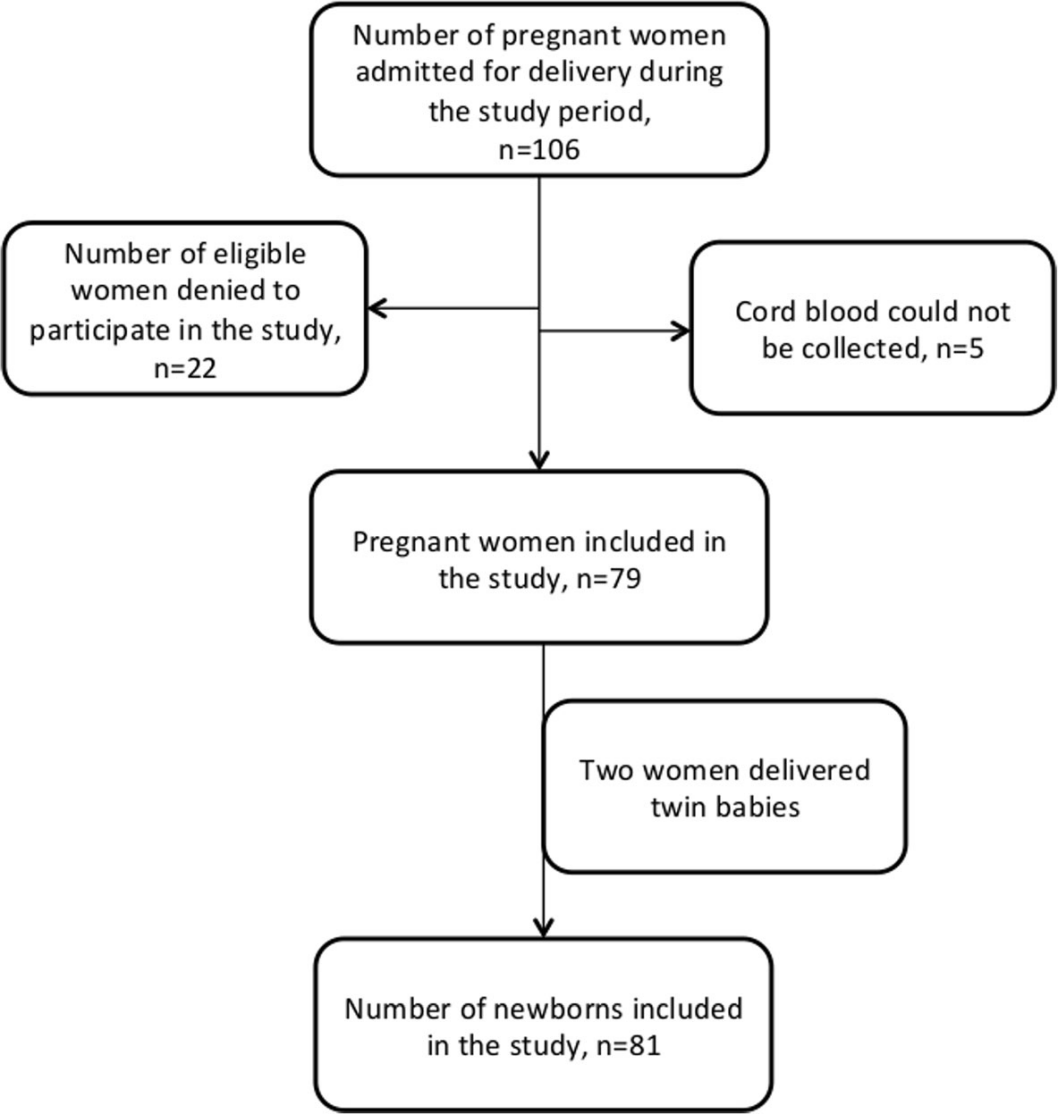
Flow chart of enrollment of pregnant women for the study

There were 106 pregnant women admitted to the hospital for delivery during in the study period out of which 79 pregnant women were included in the study; two had twin babies, so the total numbers of babies in the study were 81 (Fig. 1). The mean age of mothers was 26.7 years with a minimum of 18 years and the maximum of 38 years. General characteristics of the mothers are shown in Table 2. 97.5% (77/79) of the mothers took iron supplementation during the pregnancy, 69.6% (55/79) took folic acid, and 97.5% (77/79) took calcium supplementation. General characteristics of the babies are shown in Table 3. One fifth (19.7%) of the babies were low birth weight (< 2.5 kg) and 7.6% (6/81) were preterm.

**Table 2:** General characteristics of pregnant women admitted for delivery to Siddhi Memorial Hospital

Characteristics	Frequency, *n* = 79 (%)
Age [mean (SD)], years	26.7 (4.7)
Height [mean (SD)], cm	151.9 (10.7)
Weight [mean (SD)], kg	65.2 (9.1)
Duration of marriage [mean (SD)], years	4.5 (3.9)
Education	
Masters	7 (8.9)
Bachelor	21 (26.6)
Intermediate	23 (29.1)
SLC	5 (6.3)
School	23 (29.1)
Occupation	
Housewife	47 (59.5)
Service Holder	23 (29.1)
Business	7 (8.8)
Farmer	1 (1.3)
Student	1 (1.3)
Dietary habit	
Vegetarian	3 (3.8)
Non -vegetarian	76 (96.2)
Blood group	
A+	32 (40.5)
0+	21 (26.6)
B+	16 (20.2)
AB+	8 (10.1)
A-	1 (1.3)
B-	1 (1.3)
No. of previous deliveries	
0	46 (58.2)
1	27 (34.2)
2	4 (5.1)
3	2 (2.5)
Gestational age [mean (SD)], weeks	38.4 (1.7)
Type of delivery	
Vaginal delivery	35 (44.3)
Emergency caesarian section	32 (40.51)
Elective caesarian section	12 (15.2)

**Table 3:** General characteristics of newborn babies

Characteristics	*n* = 81 (%)
Sex	
Male	44 (54.3)
Female	37 (45.7)
Birth weight, kg	
< 2.5	16 (19.7)
2.5–4	65 (80.2)
Birth length [mean (SD)], cm	48.5 (3.4)
Head circumference [mean (SD)], cm	33.7 (1.7)
Gestation	
Preterm (< 37 weeks)	6 (7.6)
Term (37 to 42 weeks)	73 (92.4)
Outcome	
Admitted for observation	9 (11.1)
Normal	72 (88.9)

The prevalence of vitamin D deficiency [< 20 ng/ml] and insufficient vitamin D level [20–30 ng/ml] among pregnant women at the time of delivery were 81% (64/79) and 11.39% (9/79), respectively. Similarly, 67% (53/79) of the pregnant mothers had lower serum calcium level than the desired value [8.4–10.2 mg/dl]. 35.8% (29/81) and 64.2% (52/81) of the babies were found to have vitamin D deficiency [< 20 ng/ml] and calcium deficiency [< 9 mg/dl] in their cord blood, respectively (Table 4).

**Table 4:** Summary of serum vitamin D and calcium levels in mothers and newborn babies

Mothers		Baby (cord blood)	
Range	*n* = 79 (%)	Range	*n* = 81 (%)
Vitamin D (ng/ml)			
< 20	64 (81)	< 20	29 (35.8)
20–29	9 (11.4)	20–29	30 (37)
30–100	6 (7.6)	30–100	22 (27.2)
> 100	0 (0)	> 100	0 (0)
Calcium (mg/dl)			
< 8.4	53 (67)	< 9	52 (64.2)
8.4 – 10.2	26 (33)	9–11.5	29 (35.8)
> 10.2	0 (0)	> 11.5	0 (0)

**Fig. 2:**
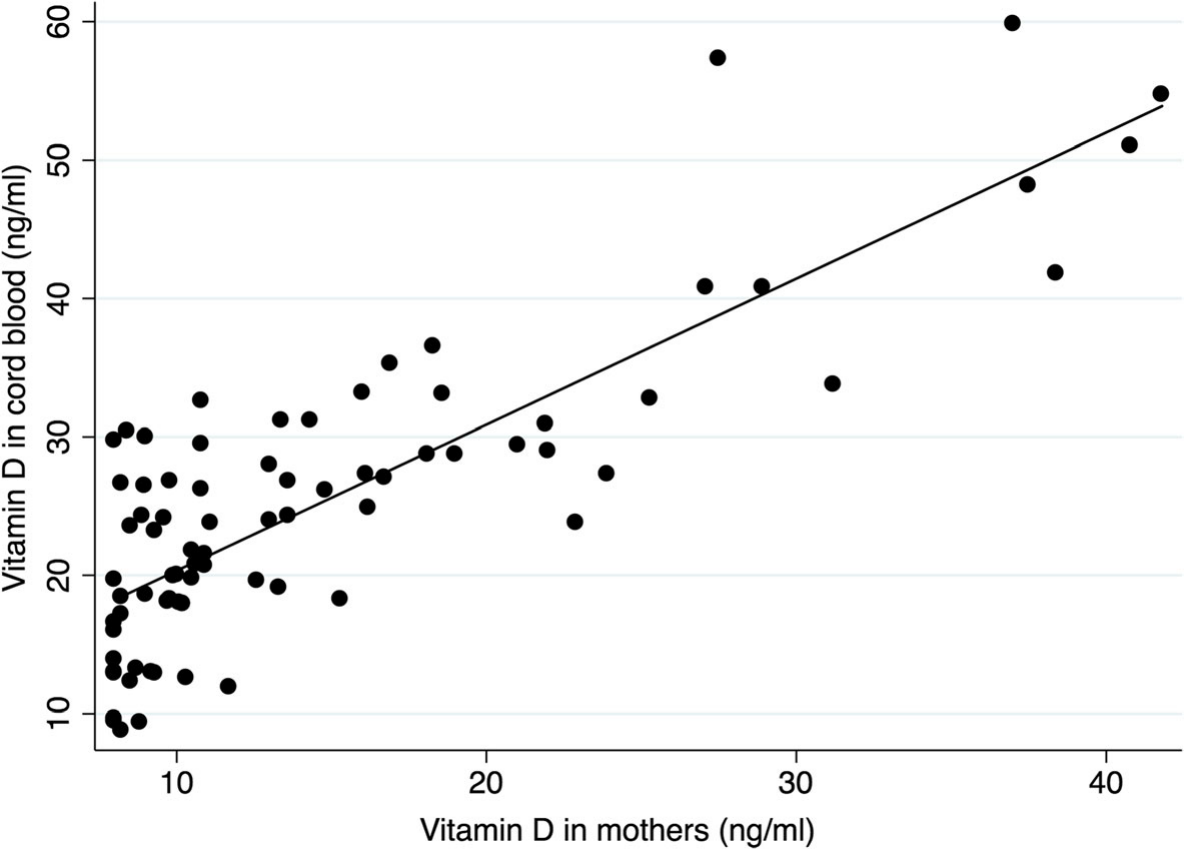
Linear relationship between serum vitamin D in mother and cord blood (newborn)

Serum vitamin D levels in mothers and babies were plotted in a scatter plot (Fig. 2). The association of the vitamin D levels in mothers with babies was explored by using the linear regression model; the coefficient of regression was 1.05 (95% CI 0.89–1.21; *P* < 0.001) and 1.04 (95% CI 0.88–1.02; *P* < 0.001) when adjusted with sex, birth weight and gestational age. Among the pregnant women and babies with vitamin D level < 30 ng/ml, the coefficient of regression was 0.84 (95% CI 0.52–1.17; *P* < 0.001) and 0.83 (95% CI 0.51–1.15; *P* < 0.001) when adjusted with the covariates. Similarly, the relationship between serum calcium level in mothers and in babies was explored. The coefficient of regression was 0.52 (95% CI 0.29–0.76; *P* < 0.001), and 0.53 (95% CI 0.29–0.76) when adjusted for the covariates. Among the pregnant mothers with serum calcium < 8.4 mg/dl and babies with cord blood level < 9.0 mg/dl, the coefficient was 0.54 (95% CI 0.22–0.86; *P* = 0.001) and 0.53 (95% CI 0.21–0.85; *P* = 0.002) when adjusted with the covariates.

## Discussion

This study showed a high prevalence of vitamin D deficiency and a high prevalence of lower than desired level of calcium among pregnant women and their newborn babies in Bhaktapur, Nepal. The levels of the micronutrients in mothers had a linear relationship with those of newborn babies. To our knowledge, this is the first study specifically looking into the relationship of vitamin D level in pregnant women and their newborn babies in Nepal. Few studies showed high a prevalence of vitamin D deficiency in general population [15, 16]. In a study conducted in Nepal vitamin D deficiency (< 20 ng/ml) was observed in 81% of the pregnant women and serum calcium level was less than the desired value in 67% of the mothers [17].

Vitamin D deficiency is observed worldwide in all age groups [13–18]. Deficiency during pregnancy and childbirth poses more threat to health than in other periods of life [4–6, 19, 20]. This can be prevented by nutrient supplementation. South Asia is considered to be high-risk area for vitamin D deficiency because of its geographical location and dark skin color of the people [10, 13]. A study done in a rural part of North India showed vitamin D deficiency (< 20 ng/ml) in 88.6% of adolescent girls and 74% of pregnant women [21]; similarly another study in the same region found 85% of pregnant women and 95% of their newborn babies had vitamin D deficiency with a positive correlation between maternal and cord blood vitamin D level [12]. Many studies have shown a positive correlation between maternal and babies’ vitamin D levels as vitamin D is transferred from mother to fetus via placenta [20, 22–24].

Pregnant women in Nepal are regularly supplemented with calcium and vitamin D3 tablets containing 500 mg of elemental calcium and 250 IU of vitamin D3 (cholecalciferol) after first trimester of pregnancy. The WHO has recommended 1 to 2 g of elemental calcium during pregnancy and child birth, but not a regular vitamin D supplementation during normal pregnancy [24–27]. However, the Royal College of Obstetricians and Gynecologists (RCOG) of the United Kingdom and the American College of Obstetricians and Gynecologists (ACOG) has recommended at least 400 IU of vitamin D3 supplementation during pregnancy [2, 28]. As the region is prone to vitamin D deficiency and studies have shown high prevalence of deficiency in the region, it should be considered to follow the recommendation of these Royal Colleges to provide at least 400 IU of vitamin D3 as a regular supplementation to Nepalese pregnant women.

Serum calcium is an important marker of vitamin D activity. An adequate amount of vitamin D is required for maintenance of calcium homeostasis [8, 28]. There is a risk of calcium deficiency when there is vitamin D deficiency. Positive correlation between maternal serum vitamin D and calcium with that of cord blood vitamin D and calcium level has been documented [1, 4, 12]. Determining vitamin D and calcium level in pregnant mothers can help us to predict their levels in newborns. In our study, the linear relationship is stronger for the vitamin D levels as compared to the calcium levels (adjusted coefficient of linear regression was 1.04 versus 0.53).

The study has some limitations. The study site was a non-governmental hospital; the services were not free of cost as that of governmental hospital, so the study population tended to be pregnant women of middle or higher socio-economic status. Due to this, the prevalence of vitamin D deficiency could have been underestimated. As the study was carried over only for 6 months the effects of seasonal variations could not be studied.

## Conclusion

We have found a high prevalence of vitamin D deficiency among the pregnant women and their newborn babies in Nepal. Further large studies are needed to explore the possible causes of vitamin D deficiency. Regular adequate supplementation of vitamin D and calcium are needed during pregnancy.

## Abbreviations

25(OH)D2/D3: 25 – Hydroxyvitamin D2/D3
AAP: American Academy of Pediatrics
ACOG: American College of Obstetrics and Gynecologists
CDC: Center for Disease Control
NHRC: Nepal Health Research Council
o-CPC: o-Cresolphthalein Complexone
RCOG: Royal College of Obstetricians and Gynecologists
SD: Standard Deviation
